# The Relationship between Dental Follicle Width and Maxillary Impacted Canines' Descriptive and Resorptive Features Using Cone-Beam Computed Tomography 

**DOI:** 10.1155/2017/2938691

**Published:** 2017-11-01

**Authors:** İlhan Metin Dağsuyu, Rıdvan Okşayan, Fatih Kahraman, Mehmet Aydın, İbrahim Şevki Bayrakdar, Mehmet Uğurlu

**Affiliations:** ^1^Department of Orthodontics, Faculty of Dentistry, Eskişehir Osmangazi University, Eskişehir, Turkey; ^2^Department of Oral and Maxillofacial Radiology, Faculty of Dentistry, Eskişehir Osmangazi University, Eskişehir, Turkey; ^3^Department of Orthodontics, Faculty of Dentistry, Atatürk University, Erzurum, Turkey

## Abstract

**Objectives:**

To assess the relationship between dental follicle width and maxillary impacted canines' descriptive and resorptive features with three-dimensional (3D) cone-beam computed tomography (CBCT).

**Methods:**

The study comprised 102 patients with cone-beam computed tomography 3D images and a total of 140 impacted canines. The association between maxillary impacted canine dental follicle width and the variables of gender, impaction side (right and left), localization of impacted canine (buccal, central, and palatal), and resorption of the adjacent laterals was compared. Measurements were analyzed with Student's *t*-test, Kruskal-Wallis test, and Mann–Whitney *U* statistical test.

**Results:**

According to gender, no statistically significant differences were found in the follicle size of the maxillary impacted canine between males and females (*p* > 0.05). Widths of the follicles were determined for the right and left impaction sides, and no statistically significant relation was found (*p* > 0.05). There were statistically significant differences between root resorption degrees of lateral incisors and maxillary impacted canine follicle width (*p* < 0.05). Statistically significant higher follicle width values were present in degree 2 (mild) resorption than in degree 1 (no) and degree 3 (moderate) resorption samples (*p* < 0.05).

**Conclusions:**

No significant correlation was found between follicle width and the variables of gender, impaction side, and localization of maxillary impacted canines. Our study could not confirm that increased dental follicle width of the maxillary impacted canines exhibited more resorption risk for the adjacent lateral incisors.

## 1. Introduction

Maxillary canines have a great importance in facial aesthetics and dental occlusion. Prevalence rate of maxillary impacted canines is reported to be from 1% to 5% and has a high rate of occurrence in females [1, 2]. The impaction of maxillary canines may cause resorptions in adjacent teeth, cystic lesions, tumors, and discrepancies in dental arch length and width [3]. Compared to other teeth, the maxillary canines have the longest eruption path between their formation region and the final occlusal position in the dental arch [4]. This may explain why maxillary canines are known as the second most frequently impacted teeth after the third molars.

Conventional two-dimensional (2D) images show errors and unsatisfactory information in impacted tooth evaluation. With the development of three-dimensional (3D) imaging, conventional radiography aided maxillary impacted canine studies are starting to be done again with the use of computed tomography (CT). Recently, cone-beam computed tomography (CBCT) has become an alternative to conventional CT in diagnosis and treatment planning of impacted teeth [5, 6]. Also, CBCT has many advantages, it is reliable, reduces distortion, costs less, and allows a diminished radiation dose [7]. Bjerklin and Ericson found that, after 3D assessment of maxillary impacted canines, orthodontic treatment plans were changed in nearly half of the cases [8].

Tooth eruption is related to the dental follicle and depends on the environmental, metabolic, and cellular activities around it [9]. Researchers reported that dental follicle cells change to other cells, such as cementoblasts, osteoblasts, and periodontal ligament cells [10, 11]. In addition to this, follicle dimensions and tooth eruption may be differentiated by hormones and growth factors [12, 13]. Ericson et al. revealed that the maxillary canine dental follicle will frequently expose the root of the laterals and centrals without resorption of the root's hard tissues and as a result a normal tooth eruption period is observed [14]. Ericson and Bjerklin studied the shape and size of dental follicles in ectopically and normally erupting maxillary canines using CT [9]. In another study, Ericson et al. researched the relationship between resorption in permanent incisor roots and canine dental follicles with CT [14]. According to some CT studies, the size difference of maxillary impacted canine dental follicles may be responsible for the resorption in adjacent teeth, especially in incisors [15, 16]. In a limited number of CBCT studies, only the follicle width was classified as normal or wider than normal [3, 7]. Unlike other studies, in our study, maxillary impacted canine follicle width (mm) was compared between all resorption degrees in adjacent lateral incisors by CBCT.

The purpose of this study was to determine whether there is a relationship between maxillary impacted canine follicle width and gender, localization, impaction side, and adjacent teeth resorption of impacted canines with CBCT.

## 2. Subjects and Methods

This retrospective study was based on the 3D CBCT records of 102 patients (43 males, 59 females; mean age 16.25 +/− 6.31) and a total of 140 impacted canines. Patients had been referred to the orthodontic clinic for consultation or malocclusion treatment. The investigation protocols were approved by the Clinical Research Ethical Committee of Eskişehir Osmangazi University (80558721/G-131). As previously mentioned in the literature, maxillary canine impaction was identified if the root formation was completed or the other side canine was completely erupted to the occlusal plane [17]. Samples were excluded from the study due to the presence of systemic bone disease, orthodontic treatment history, pathology around the impacted canine, and craniofacial syndromes.

All CBCT images were taken with the same CBCT device in a standing position (Promax 3D. Mid; Planmeca, Helsinki, Finland). Parameters included tube voltage of 94 kVp, tube current of 14 mA, and a scanning time of 27 seconds. The CBCT images were evaluated in all three planes (sagittal, axial, and coronal) by a single investigator (F.K.). Simplant O&O (Materialise, Leuven, Belgium) dental software was used for linear and diagnostic 3D CBCT measurements.

The samples' descriptive information (age, gender) was recorded. Impaction side (right or left), localization (buccal, central, or palatal), and root resorption levels in lateral teeth variables related to follicle size were evaluated on the CBCT images.

**Figure 1:**
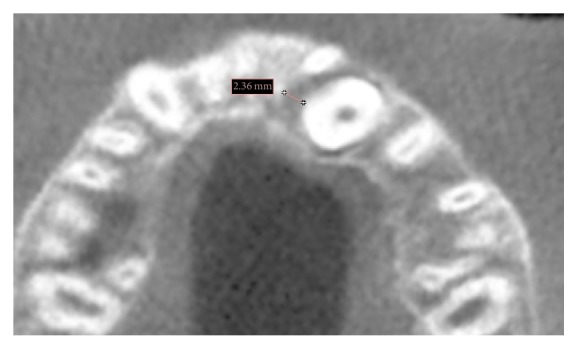
Width measurement of the follicle in the axial CBCT image.

Follicle width was measured at the furthest distance from the maxillary impacted canine crown to the periphery of the follicle in the axial CBCT image (Figure 1) [9]. The resorption features of lateral incisors adjacent to maxillary impacted canine dental follicles were evaluated. A resorption classification is identified with numbers 1 to 4, as mentioned in the literature [18]. Degree 1 indicates absence of resorption; degree 2 indicates mild resorption (up to half the dentine thickness); degree 3 indicates moderate resorption (very close to the pulp; but the pulp is covered with unbroken dentin); and degree 4 indicates severe resorption (the pulp is exposed).

Data were analyzed with MedCalc statistical software (MedCalc Software, Windows V. 17.1, Broekstraat, Mariakerke, Belgium). To assess the normality, the Kolmogorov-Smirnov test was used. Student's *t*-test for independent samples was applied to analyze the gender and impaction side variables. Resorption degree and localization comparison to follicle width were analyzed with the Kruskal-Wallis and Mann–Whitney *U* tests. All statistical analyses were performed at the 0.05 significance level.

## 3. Results

The dental follicle width of the maxillary impacted canines was measured at 3.13 +/− 1.20 mm for all 140 impacted canines. The evaluation of the follicle width of maxillary impacted canines according to gender is 3.27 +/− 1.21 mm in males and 3.08 +/− 1.20 mm in females. In terms of the follicle width variable, no statistically significant differences were found between males and females (*p* > 0.05). Sixty-eight maxillary impacted canines were located on the left side, and 72 maxillary impacted canines were located on the right side. No significant differences in the width of the follicles were determined for the right and left impaction sides (*p* > 0.05).  Table 1 shows the evaluation of whether the localization (buccal, central, and palatal) of the maxillary impacted canine in the jaw is related to follicle size of the maxillary impacted canine. Respectively, follicle widths of the maxillary impacted canines were 3.26 +/− 1.51 mm for buccal, 3.04 +/− 1.02 mm for central, and 3.13 +/− 1.17 mm for palatal localization. According to the results, no statistically significant differences were found between the follicle width of the maxillary impacted canines and the localization of the impacted canines (*p* > 0.05).

The resorption degrees of 140 lateral incisor teeth (72 in right side, 68 in left side) were compared with the maxillary impacted canine follicle width. A total of 63 maxillary lateral incisors showed no resorption (follicle width: 2.97 +/− 1.21), 52 showed mild resorption (follicle width: 3.51 +/− 1.19), 15 lateral incisors had moderate resorption (follicle width: 2.60 +/− 0.60), and 10 maxillary lateral teeth showed severe resorption (follicle width: 2.91 +/− 1.35). There were statistically significant differences between root resorption degrees of lateral incisors and maxillary impacted canine follicle widths (*p* < 0.05) (Table 2). More statistically significant higher follicle width values were present in degree 2 (mild) resorption than in degree 1 (no) and degree 3 (moderate) resorption samples (*p* < 0.05).

## 4. Discussion

This retrospective study provides a comparison of the maxillary impacted canine follicle width and gender, localization, impaction side, and adjacent teeth resorption with CBCT. It has been reported that there are two common impaction theories for maxillary canines. The first is the guidance theory of canine impaction: when the lateral incisors are congenitally absent or peg-shaped or have a development deficiency, the maxillary canines lose their dental guidance, and failure is indicated in the normal eruption path. The second is the genetic theory: if maxillary canine impaction is related to genetic control, it can be seen bilaterally at a high percentage, revealing a relation with palatal impaction [19]. The impacted maxillary canines may need to be controlled during the process of eruption. Therefore, CBCT is a valuable imagining method in situations where there is a risk for resorption in adjacent teeth, especially in cases of maxillary impacted canines.

In our study, for all samples, the dental follicle width measured was 3.13 mm, and, according to the literature, our results were in the normal range. Walker et al. found similar average follicle width values (3.6 mm) in their impacted canine study, and they revealed that there was no relationship between follicle size and impaction of the canine [20].

According to our results, there was no relationship between gender and follicle width in subjects with maxillary impacted canines. Similar to our study results, Ericson and Bjerklin revealed that there was a large variation in follicle width in same gender groups, but no significant differences between males and females were found for the normally and the ectopically erupting canines [9]. Right side follicles were wider than left side maxillary impacted canine follicles, but there was no relationship between follicle width and side of impaction in maxillary impacted canines.

The maxillary incisor root resorption related to maxillary impacted canines was found to have a 12% prevalence and was four times higher in females than in males [21]. In our study, the largest statistically significant dental follicles of maxillary impacted canines were found to be more common in mild resorption cases (3.51 +/− 1.19 mm) in the adjacent laterals. In the literature, another conventional CT study found results similar to our investigation; they revealed that no relationship existed between dental follicle width and resorption of the permanent incisors [14]. Before the development of CBCT, Ericson and Kurol studied intraoral radiographs for assessment of the thickness of the impacted maxillary canine dental follicle, and they found 78% of the cases had a normal follicle thickness and 22% had exceeded a 3 mm follicle thickness. In addition, they did not find any association between the resorption and thickness of the canine follicle [21]. However, with the use of CBCT, it is now possible to detect regions of resorption that are not diagnosable on intraoral radiographs. Lofthag-Hansen et al. reported that CBCT provides more information about the size of the follicle and resorptions in adjacent teeth [22].

In terms of the prognosis of orthodontic treatment with extraction, it may be that adjacent lateral incisors with severe resorption can be extracted in a place of the first permanent premolar. Moreover, it is important to note that, in our literature review, we have not yet encountered any 3D CBCT research on the determination of the follicle width in different adjacent lateral resorption degrees, so our study is apparently the first in this respect.

The relationship between impacted canine localization (buccal, central, and palatal) and ectopically erupted canine follicle width was analyzed in our study. Buccally positioned impacted canine follicles were wider than those positioned palatally and centrally, respectively, but widths of the follicles of the impacted canines showed no statistically significant results between groups. Ericson and Bjerklin stated that buccally and apically displaced canines had wider follicles than normally positioned canines. They also revealed that palatally displaced canines had similar follicle size as normally positioned canines [9].

The use of CBCT in orthodontics improves our understanding of maxillary impacted canines' angular, linear, and resorption features. The results of our study may assist clinicians in understanding that it is not possible to predict the presence of resorptive features of maxillary impacted canines to adjacent lateral incisors by observing the follicle width of ectopically erupted canines. The resorption events of maxillary impacted canines may be related to the active eruption pressure and cellular activities of environmental tissues in the eruption process. Neighboring anatomical structures near the follicle can have an influence on the dental follicle size and shape. Further research is needed to compare the follicle width with other variables, such as genetic components, bone quality, hormones, growth factors, and follicle shape (symmetrical or asymmetrical) on CBCT images within a larger sample size in maxillary impacted canine cases.

## 5. Conclusions

Within the limitations of this retrospective CBCT study, the results provide five main conclusions:

(1) There was no significant correlation between gender and follicle width in maxillary impacted canines; this suggests that follicle width is independent of the subject's gender.

(2) No significant association exists between the side of the maxillary canine impaction and follicle size.

(3) Follicle width of the impacted canines showed no statistically significant results for the different localizations of maxillary impacted canines (buccal, central, and palatal).

(4) CBCT images may help clinicians for prediction of maxillary canine impaction, diagnosing resorptions in adjacent laterals, and treatment of maxillary impacted canines.

(5) Larger dental follicles of maxillary impacted canines were more common in mild resorption cases in adjacent laterals. According to this result, our study could not confirm that increased dental follicle width of the maxillary impacted canines exhibited more resorption risk on adjacent lateral incisors.

## Figures and Tables

**Table 1:** Statistical results of dental follicle width according to maxillary canine localization.

Localization	Number of impacted canines (*n* = 140)	Mean of dental follicle width (mm) +/− s.d.	*p*	Buccal-central	Buccal-palatal	Central-palatal
Buccal maxillary canine impaction	25	3,26 +/− 1,51	0.902	0.896	0.978	0.613
Central maxillary canine impaction	39	3,04 +/− 1,02				
Palatal maxillary canine impaction	76	3,13 +/− 1,17				

s.d.: standard deviation. Statistically significant difference level is *p* < 0.05.

**Table 2:** Statistical results of dental follicle width according to resorption features in both right and left laterals.

Resorption degree	Number of right-left laterals (*n* = 140)	Mean of dental follicle width (mm) +/− s.d.	*p*	Degrees 1-2	Degrees 1–3	Degrees 1–4	Degrees 2-3	Degrees 2–4	Degrees 3-4
Degree 1 (none)	63	2.97 +/− 1.21	0.012^*∗*^	0.010^*∗*^	0.385	0.791	0.003^*∗*^	0.130	0.955
Degree 2 (mild)	52	3.51 +/− 1.19							
Degree 3 (moderate)	15	2.60 +/− 0.60							
Degree 4 (severe)	10	2.91 +/− 1.35							

s.d.: standard deviation. ^*∗*^Statistically significant difference level is *p* < 0.05.
